# Heterochromatin and microsatellites detection in karyotypes of four sea turtle species: Interspecific chromosomal differences

**DOI:** 10.1590/1678-4685-GMB-2020-0213

**Published:** 2020-12-02

**Authors:** Caroline Regina Dias Machado, Camila Domit, Marcela Baer Pucci, Camilla Borges Gazolla, Larissa Glugoski, Viviane Nogaroto, Marcelo Ricardo Vicari

**Affiliations:** 1 Universidade Federal do Paraná, Centro Politécnico, Departamento de Genética, Programa de Pós-Graduação em Genética, Curitiba, Ponta Grossa, PR, Brazil.; 2 Universidade Federal do Paraná, Laboratório de Ecologia e Conservação, Pontal do Paraná, PR, Brazil.; 3 Universidade Nove de Julho, Departamento de Saúde II, Bauru, SP, Brazil.; 4 Universidade Federal de São Carlos, Programa de Pós-Graduação em Genética Evolutiva e Biologia Molecular, São Carlos, SP, Brazil.; 5 Universidade Estadual de Ponta Grossa, Departamento de Biologia Estrutural, Molecular e Genética, Ponta Grossa, PR, Brazil.

**Keywords:** Cheloniidae, chromosomal rearrangements, Cryptodira, endangered species, repetitive DNAs

## Abstract

The wide variation in size and content of eukaryotic genomes is mainly attributed to the accumulation of repetitive DNA sequences, like microsatellites, which are tandemly repeated DNA sequences. Sea turtles share a diploid number (2n) of 56, however recent molecular cytogenetic data have shown that karyotype conservatism is not a rule in the group. In this study, the heterochromatin distribution and the chromosomal location of microsatellites (CA)_n_, (GA)_n_, (CAG)_n_, (GATA)_n_, (GAA)_n_, (CGC)_n_ and (GACA)_n_ in *Chelonia mydas, Caretta caretta, Eretmochelys imbricata* and *Lepidochelys olivacea* were comparatively investigated. The obtained data showed that just the (CA)_n_, (GA)_n_, (CAG)_n_ and (GATA)_n_ microsatellites were located on sea turtle chromosomes, preferentially in heterochromatic regions of the microchromosomes (mc). Variations in the location of heterochromatin and microsatellites sites, especially in some pericentromeric regions of macrochromosomes, corroborate to proposal of centromere repositioning occurrence in Cheloniidae species. Furthermore, the results obtained with the location of microsatellites corroborate with the temperature sex determination mechanism proposal and the absence of heteromorphic sex chromosomes in sea turtles. The findings are useful for understanding part of the karyotypic diversification observed in sea turtles, especially those that explain the diversification of Carettini from Chelonini species.

## Introduction

Sea turtles (Testudines: Cryptodira) are grouped into Dermochelyidae and Cheloniidae families and only seven living species (Pritchard, 1997). Dermochelyidae is monotypic, represented by *Dermochelys coriacea* (leatherback sea turtle), and is the sister-taxon to a clade comprising all other extant sea turtles (Bowen and Karl, 1996; Dutton *et al*., 1996; Parham and Fastovsky, 1997; Iverson *et al*., 2007). Molecular phylogenetic studies supported recognition of two tribes in Cheloniidae: (*i*) Chelonini grouping *Natator depressus* (flatback sea turtle) and *Chelonia mydas* (green sea turtle) and; (*ii*) Carettini grouping *Lepidochelys olivacea* (olive ridley sea turtle), *Lepidochelys kempii* (Kemp’s ridley sea turtle), *Eretmochelys imbricata* (hawksbill sea turtle) and, *Caretta caretta* (loggerhead sea turtle) (Iverson *et al*., 2007; Naro-Maciel *et al*., 2008). 

All sea turtle species show different levels of threat of extinction and are considered flag species for the conservation of biodiversity (IUCN, 2020) since numerous threats affect the populations maintenance (Lara-Ruiz *et al*., 2006; Proietti *et al*., 2014; Arantes *et al*., 2020). In addition, chromosomal studies in turtles demonstrated great karyotypic diversification among evolutionary lineages, making the cytogenetic knowledge important for recognition of species diversity and conservation (Valenzuela and Adams, 2011; Montiel *et al*., 2016; Rovatsos *et al*., 2017; Cavalcante *et al*., 2018; Lee *et al*., 2019; Deakin and Ezaz, 2019; Clemente *et al*., 2020; Viana *et al*., 2020). 

Testudines cytogenetic studies demonstrated karyotypes composed of macrochromosomes and a variable number of microchromosomes (mc), a characteristic shared with birds and squamate reptiles (Olmo, 2008; Pokorná *et al*., 2011a; Montiel *et al*., 2016). In turtles, three karyotypic groups were proposed according to the diploid number (2n): (*i*) high 2n (60 - 68 chromosomes) and high amount of mc; (*ii*) intermediate 2n (50 - 56 chromosomes) and relatively low amount of mc and; (*iii*) low 2n (26 - 28 chromosomes) and without mc (Ayres *et al*., 1969; Barros *et al*., 1976; Sites *et al*., 1979). However, genomic and cytogenetic studies using comparative analyses of chromosomal markers are scarce in Testudines species (Iannucci *et al*., 2019; Cavalcante *et al*., 2018, 2020a, b).

The wide variation of 2n (26 - 68 chromosomes) observed in turtles implies that their genomes have been deeply reorganized (Noleto *et al*., 2006; Valenzuela and Adams, 2011; Montiel *et al*., 2016; Noronha *et al*., 2016; Cavalcante *et al*., 2018, 2020a, 2020b; Clemente *et al*., 2020). In this sense, the characterization of repetitive DNA sequences present in heterochromatin sites allow us to understand chromosomal rearrangements in some species of the group (Cavalcante *et al*., 2018, 2020a, 2020b). In turtles, as well as in sister groups, heterochromatin is located in the pericentromeric region of most macrochromosomes and some mc (Nishida *et al*., 2013; de Oliveira *et al*., 2017; Cavalcante *et al*., 2018; Barcellos *et al*., 2019; Viana *et al*., 2020). In crocodilians, which do not have mc, the heterochromatic regions were also preferentially located in the pericentromeric regions (Amavet *et al*., 2003; Kawagoshi *et al*., 2008).

Heterochromatin is usually composed by an enriched repetitive DNA segment of satellite DNAs (Sumner, 2003). Satellite, minisatellites and microsatellites DNAs were initially classified according to both the length of the whole repeat cluster and the size of the repetitive unit (Tautz, 1993). Although controversy still exists, the term satellite DNA has been applied to any tandem repetitive sequence which is present in blocks of hundreds to thousands of units and which are located in heterochromatin sites regardless of unit size (Garrido-Ramos, 2015). For instance, microsatellites are 1-6 nucleotide units tandemly repeated and can be found accumulated as a part constituent of heterochromatin (Kubat *et al*., 2008; Schemberger *et al*., 2019; Viana *et al*., 2020; Zattera *et al*., 2020) or located dispersed in euchromatic chromosomes regions (Tóth *et al*., 2000; Ruiz-Ruano *et al*., 2015). Their location on chromosomes can be species-specific or present a similar distribution pattern in close relationship species groups (Tóth *et al*., 2000; Ziemniczak *et al*., 2014; Pucci *et al*., 2016). 

**Figure 1 -:**
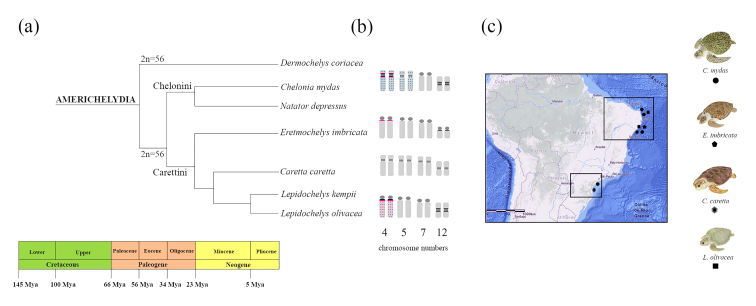
Phylogeny, sampled area, and details of chromosome changes in four Cheloniidae species: In (a) phylogeny and ancestral reconstruction of sea turtle species adapted from Pereira *et al*. (2017); In (b), representative idiograms of the pairs 4, 5, 7 and 12 involved in chromosomal changes among sea turtle species; and (c) partial map of South America showing sea turtles sampled in different places in the Brazil and referred by geometric forms, *C. mydas* (circle), *E. imbricatta* (pentagon), *C. caretta* (asterisk), and *L. olivacea* (square). Images of sea turtles (Source: Projeto Tamar).

Based on phylogenetic analysis in Testudines, Montiel *et al*. (2016) proposed that the putatively ancestral condition for Dermochelyidae and Cheloniidae species is a 2n of 56 chromosomes (Figure 1). In previous studies, all sea turtle species shared 2n = 56 and their karyotypes were considered identical (Bickham *et al*., 1980; Bickham and Carr, 1983; López *et al*., 2008; Fukuda *et al*., 2014). However, a recent comparative cytogenetic study showed species-specific differences in chromosomal morphology, G-banding patterns, besides interstitial telomeric sites (ITS) occurrence, among *C. mydas*, *L. olivacea*, *E. imbricata* and, *C. caretta*, which ruled out the proposal of conserved structure of the macrochromosomes in Cheloniidae (Machado *et al*., 2020). Here, chromosomal data of sea turtle species from Cheloniidae that occur in the Brazilian coast: *C. mydas*, *C. caretta*, *E. imbricata*, and *L. olivacea* were comparatively analyzed, aiming to describe the heterochromatin and microsatellites chromosomal locations, inferring its relations with the interspecific karyotype diversity.

## Material and Methods

### Sampling and chromosome preparation

*Chelonia mydas*, *C. caretta*, *E. imbricata* and *L. olivacea* were cytogenetically compared. The biological samples were obtained captive or wild animals in different areas of the Brazilian coast (Figure 1; for details, see Table S1). Fifty sea turtles were sampled and karyotyped: (*i*) *C. mydas* (N = 27; one female and 26 juveniles), (*ii*) *C. caretta* (N = 11; two males, five females and four juveniles), (*iii*) *E. imbricata* (N = 6; two females and four juveniles ), and (*iv*) *L. olivacea* (N = 6; one male and five juveniles). The collection samples were authorized by the Instituto Chico Mendes de Conservação da Biodiversidade (ICMBio, licence number 52218-7; 43433-2/3). All experimental procedures were authorized and performed following the Ethical Committee on Animal Use of the Universidade Estadual de Ponta Grossa. (Protocol: 7200/2016).

Peripheral blood was used to obtain the chromosomal preparations by temporary culture of lymphocytes method (Rodríguez *et al*., 2003). The slides containing chromosomal preparations were submitted to C-banding for constitutive heterochromatin detection (Sumner, 1972) and to fluorescence *in situ* hybridization (FISH) assays, using microsatellites probes.

### FISH

The (CA)_15_, (GA)_15_, (CAG)_10_, (GATA)_8_, (GAA)_10_, (CGC)_10_ and (GACA)_8_ microsatellites probes were directly labelled with Cy5 fluorochrome (Sigma-Aldrich, San Luis, Missouri, USA) at the 5’ end during DNA synthesis. FISH was performed according to the protocol proposed by Kubat *et al*. (2008), under ~77% stringency. Chromosomes were counterstained with 0.2 μg/mL 4’,6-diamidino-2-phenylindole - DAPI (Sigma-Aldrich) in the Vectashield mounting medium (Vector, Burlingame, CA, USA). The images were captured in CCD Olympus DP-72 camera coupled in epifluorescence microscope Olympus BX51 (Olympus, Tokyo, Japan). Twenty metaphases were analyzed per sampled individual for microsatellites signals detection.

### Karyotype organization

Chromosomes were arranged by decreasing size and centromere position, as described by Montiel *et al*. (2016). They were classified as bi-armed and one-armed (acrocentric), depending on their arm ratio, and as macrochromosomes or mc, according to Bickham *et al*. (1980) description. Microchromosomes were remarkably similar (practically indistinguishable) and thus were ordered by approximate size and chromosome marks, where possible. Representative idiograms of the karyotype organization of the four species analyzed were designed, illustrating the data obtained in the present study and those of Machado *et al*. (2020).

## Results

### Constitutive heterochromatin organization

**Figure 2 -:**
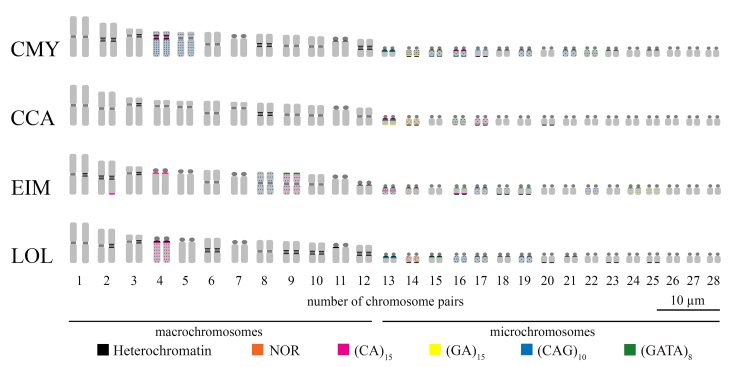
Representative idiograms of heterochromatic regions, NOR and microsatellite motifs on the chromosomes of the four sea turtle species. The species names were referred by their 3-letter acronym: *C. mydas* (CMY), *C. caretta* (CCA), *E. imbricata* (EIM) and *L. olivacea* (LOL).

**Figure 3 -:**
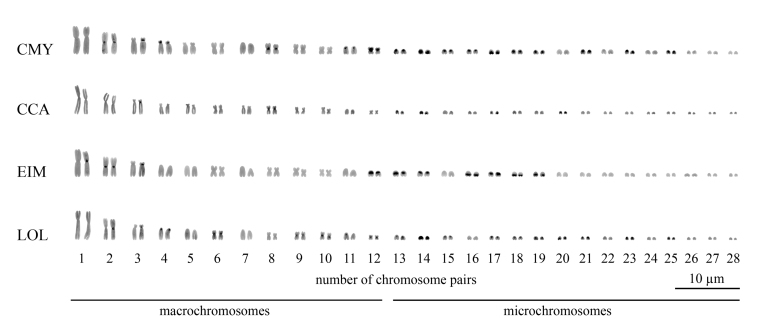
Karyotypes of sea turtle species subjected to C-banding. The species names were referred by their 3-letter acronym: *C. mydas* (CMY), *C. caretta* (CCA), *E. imbricata* (EIM) and *L. olivacea* (LOL).

**Figure 4 -:**
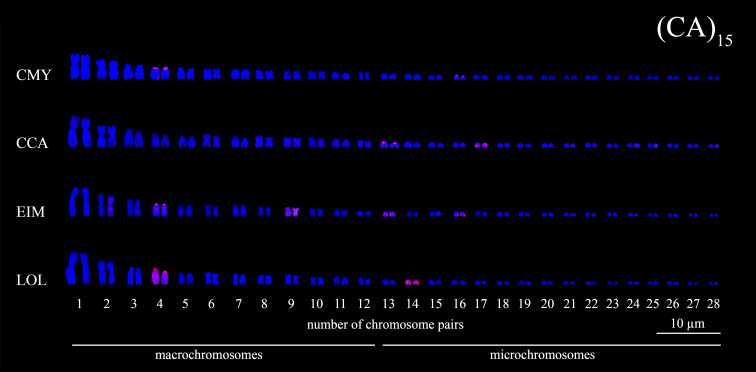
Karyotypes of sea turtle species subjected to FISH using (CA)_15_ microsatellites probes (*red signals*). The species names were referred by their 3-letter acronym: *C. mydas* (CMY), *C. caretta* (CCA), *E. imbricata* (EIM) and *L. olivacea* (LOL).

**Figure 5 -:**
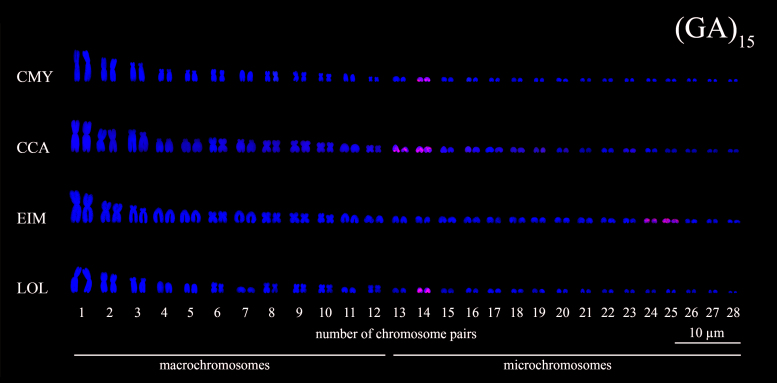
Karyotypes of sea turtle species subjected to FISH using (GA)_15_ microsatellites probes (*red signals*). The species names were referred by their 3-letter acronym: *C. mydas* (CMY), *C. caretta* (CCA), *E. imbricata* (EIM) and *L. olivacea* (LOL).

**Figure 6 -:**
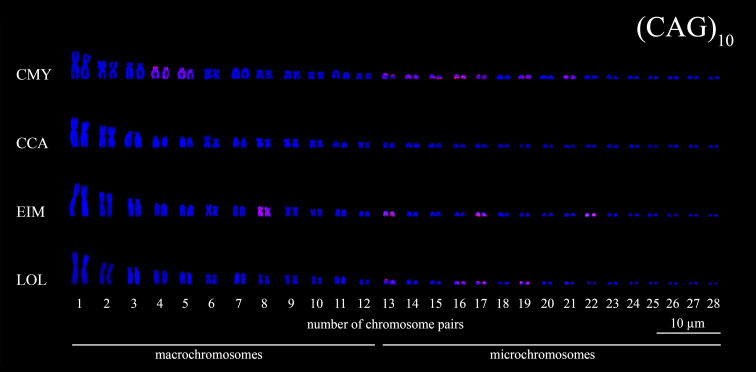
Karyotypes of sea turtle species subjected to FISH using (CAG)_10_ microsatellites probes (*red signals*). The species names were referred by their 3-letter acronym: *C. mydas* (CMY), *C. caretta* (CCA), *E. imbricata* (EIM) and *L. olivacea* (LOL).

**Figure 7 -:**
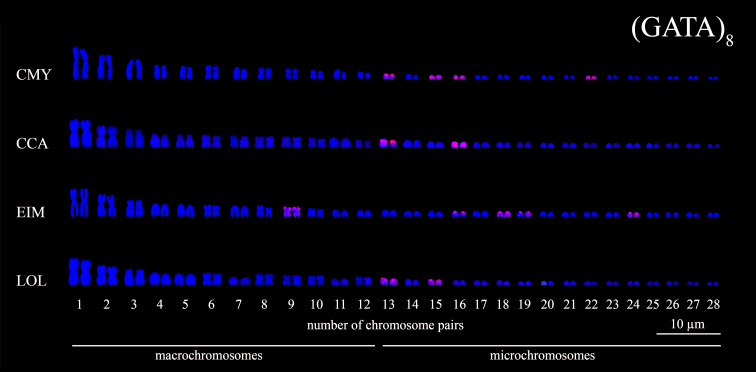
Karyotypes of sea turtle species subjected to FISH using (GATA)_8_ microsatellites probes (*red signals*). The species names were referred by their 3-letter acronym: *C. mydas* (CMY), *C. caretta* (CCA), *E. imbricata* (EIM) and *L. olivacea* (LOL).

The four species presented 2n = 56 arranged in 12 macrochromosome and 16 mc pairs and without evidences for heteromorphic sex chromosomes occurrence among all individuals sampled (Figures 2-7). *Chelonia mydas, C. caretta, E. imbricata* and *L. olivacea* showed few heterochromatic blocks in the karyotypes, as follows: (*i*) *C. mydas* showed heterochromatic blocks in the pericentromeric regions of macrochromosomes 2-4, 8, 11 and 12, besides in the pericentromeric regions of mc long arms 13-19, 21, 23 and 25 (Figures 2 and 3); (*ii*) *C. caretta* has heterochromatic blocks in the pericentromeric regions of macrochromosomes 3 and 8, as well as in the pericentromeric region of mc 13 and, in the long arm of mc 14, 17 and 20 (Figures 2 and 3); (*iii*) *E. imbricata* showed heterochromatic bands located in the pericentromeric regions of the macrochromosomes 1-3 and 12, besides in the pericentromeric regions of mc 13, 14, and 17 and, in the subterminal region of the long arm of mc 16, 18 and 19 (Figures 2 and 3); and (*iv*) in *L. olivacea* heterochromatin is located in the pericentromeric regions of the macrochromosomes 2-4, 6 and 9-12, as well as in the pericentromeric regions of mc 13, 15, 17-19 and 21 and, in the long arms of mc 14, 20, 23 and 25 (Figures 2 and 3).

### Microsatellites: distribution pattern

Distinct microsatellites signals were detected on chromosomes of *C. mydas*, *C. caretta*, *E. imbricata* and *L. olivacea* (Figures 4-7). The (GAA)_10_, (CGC)_10_ and (GACA)_8_ microsatellites probes were not detected on the chromosomes of these four species by FISH procedure. The microsatellite (CA)_n_ was located as a block in the short arms of the chromosome pairs 4 and 16 in *C. mydas* (Figures 2 and 4). *Caretta caretta* has (CA)_n_ block detected in mc 13 and dispersed in mc 17 (Figures 2 and 4). In *E. imbricata*, (CA)_n_ signals were visualized as a block in chromosome pairs 2, 4, 13 and 16, and dispersed along chromosome pair 9 (Figures 2 and 4). *Lepidochelys olivacea* has (CA)_n_ markers detected as a block in the short arms of the acrocentric pair 4, besides dispersed signals along the chromosome pairs 4 and 14 (Figures 2 and 4).

(GA)_n_ signals were detected in the mc pair 14 of *C. mydas*, in the mc pairs 13 and 14 of *C. caretta*, in the mc pairs 24 and 25 in *E. imbricata*, and in the mc 14 of *L. olivacea* (Figures 2 and 5). The microsatellite (CAG)_n_ was located as block in chromosome pairs 13, 15-17 of *C. mydas*, besides dispersed signals along chromosome pairs 4, 5, 14, 19 and 21 (Figures 2 and 6). (CAG)_n_ motifs were not detected in *C. caretta* karyotype (Figure 6), while in *E. imbricata* (CAG)_n_ signals were located dispersed along the chromosome pairs 8, 13, 17 and 22 (Figures 2 and 6). In *L. olivacea*, (CAG)_n_ signals were detected in the mc 13, 16, 17 and 19 (Figures 2 and 6). (GATA)_n_ motifs were in situ located in the mc pairs 13, 15, 16 and 22 in *C. mydas* karyotype, in the mc pairs 13 and 16 in *C. caretta*, in the chromosome pairs 9, 16, 18, 19 and 24 in *E. imbricata* and, in the mc pairs 13 and 15 of the *L. olivacea* (Figures 2 and 7).

## Discussion

Sea turtles have been studied from a cytogenetic point of view (Bickham *et al*., 1980; Bickham and Carr, 1983; López *et al*., 2008; Fukuda *et al*., 2014), but the lack of comparative karyotype studies prevents a more robust analysis of evolutionary chromosomal changes. The first comparative cytogenetic study in sea turtles was conducted by Machado *et al*. (2020) based on G-banding and *in situ* location of 18S rDNA and telomere sequences. The compared karyotypes revealed species-specific chromosomal morphology involving macrochromosomes pairs 4, 5, 7, and 12 among *C. mydas*, *C. caretta*, *E. imbricata* and *L. olivacea* (Machado *et al*., 2020) and ruled out the proposal of identical karyotypes for sea turtles. Additionally, the same four Cheloniidae sea turtles that inhabiting the Brazilian coast were here investigated and showed chromosomal differences in heterochromatin and microsatellites distribution on the karyotypes, making possible to infer new molecular mechanisms in sea turtle diversification.

The heterochromatic blocks found in macrochromosomes varied among species and demonstrated an unusual feature which only one member of the homologous bears pericentromeric heterochromatic block for macrochromosome pairs 1, 2, and 3 in some species, as also detected by Bickham *et al*. (1980) in *C. mydas*. It’s known that satellite DNA (including micro- and minisatellites) is made up of systematic in tandem repeats favoring the occurrence of ectopic recombination and genic conversion (Schweizer and Loidl, 1987; Louzada *et al*., 2020), which could led to a differential *in cis* accumulation of satellite DNA units on heterochromatin blocks, as visualized in sea turtles’ chromosomes. In other pathway, homologous recombination involving transposable elements surrounding pericentromeric region could promote substantial differences in heterochromatin block sizes (Xiao and Peterson, 2000).

Phylogenetic analyses proposed that Dermochelyidae and Cheloniidae diverged from Americhelydia ancestral lineage in the Cretaceous and that all Cheloniidae species diverged from *Dermochelys coriacea* (Iverson *et al*., 2007; Naro-Maciel *et al*., 2008; Valenzuela and Adams, 2011; Montiel *et al*., 2016; Pereira *et al*., 2017). In addition, an ancestral reconstruction demonstrated that the Chelonini tribe (*C. mydas* and *N. depressus*) split from Carettini about 34 million years ago (Mya), and in Carettini, *Eretmochelys* split from *Caretta* and *Lepidochelys* about 23 Mya (see Figure 1; Pereira *et al*., 2017). Interestingly, the distinct chromosome pairs morphologies observed among *C. mydas*, *C. caretta*, *E. imbricata* and *L. olivacea*, proposed by Machado *et al*. (2020), also had diverged in pericentromeric heterochromatin composition, mainly in the pairs 4 and 12.

Different chromosome morphologies (considering the centromere position) in a homeologue chromosome pair among phylogenetically related species may be the result of chromosomal rearrangements (Charlesworth *et al*., 1994). However, some studies suggested that the repositioning of the centromere can occur, without changes of the DNA markers along the chromosomes (Montefalcone *et al*., 1999; Ventura *et al*., 2001). The repositioning of the centromere occurs because of the emergence of a new centromere due to numerous epigenetic changes and not by relocating an existing centromere from another genomic site (Amor *et al*., 2004). Thus, since G-banding technique did not evidence the occurrence of pericentric inversions among sea turtle chromosomes (Machado *et al*., 2020), the presence vs. absence or differences in the centromeric heterochromatin content reinforce the proposal of centromeric repositioning mechanism occurrence in the diversification of some chromosomes among *C. mydas*, *C. caretta*, *E. imbricata* and *L. olivacea*.

Changes in pericentromeric heterochromatin composition among sea turtles’ chromosomes, as partially demonstrated by microsatellites in situ localization and C-banding, could have emerged to new centromeres and could have led to morphological chromosome alterations. Applying the evolutionary scenario proposed by molecular phylogenetic studies (Iverson *et al*., 2007; Naro-Maciel *et al*., 2008; Valenzuela and Adams, 2011; Montiel *et al*., 2016, Pereira *et al*., 2017), the bi-armed chromosome pairs 4 and 5 from *C. mydas* could have diverged to acrocentric in *E. imbricata* and *L. olivacea* (Figure 1)*.* Contrary to phylogenetic data that infers *C. caretta* diverged from *E. imbricata*, *C. caretta* shared chromosome pairs 4 and 5 morphology with *C. mydas*, probably indicating chromosomal homoplasy. In addition, *C. caretta* is the only turtle, among the four species studied, that have chromosome pair 7 bi-armed, indicating specific chromosomal changes in the evolutionary lineage that still need to be solved.

Following the phylogenetic proposal, the acrocentric pair 12 of *E. imbricata* diverged from a metacentric one present in *C. mydas*, since the location of a conspicuous heterochromatic block is shared between these chromosomes and G-banding recovered its probable homeology (Machado *et al*., 2020). The proposal by Machado *et al.* (2020) of partial deletion or translocation in the origin of the chromosome pair 12 in *E. imbricata* is corroborated in the present data once no chromosomal inversion or DNA repeat unit’s retraction has been detected. In this way, the chromosomal rearrangement should be a recent and specific event occurred in *E. imbricata*, once *C. caretta* and *L. olivacea* shared the metacentric pair 12 with *C. mydas* (Figure 1).

Usually, microsatellites accumulations were demonstrated in the terminal regions of vertebrate chromosomes, which seems to be a common feature due to different mechanisms that accumulate these sequences in these regions (Pokorná *et al*., 2011b; Torres *et al*., 2011; Ruiz-Ruano *et al*., 2015; Ernetti *et al*., 2019; Lee *et al*., 2019; Viana *et al*., 2020). The (CA)_n_, (GA)_n_, (CAG)_n_ and (GATA)_n_ microsatellites were reported for the first time in sea turtle’s karyotypes and demonstrated that most of them possibly make up part of the repetitive units of heterochromatin in *C. mydas, C. caretta*, *L. olivacea* and *E. imbricata*, especially in mc. Furthermore, the chromosomal location of microsatellites sequences in the pericentromeric regions for some chromosomes is common in species groups close to turtles, such as birds and crocodilians (Rao *et al*., 2010; Nishida *et al*., 2013; Matsubara *et al*., 2016; de Oliveira *et al*., 2017; Kretschmer *et al*., 2018; Barcellos *et al*., 2019). In this study, only the (CA)_15_ repetition was observed in pericentromeric region for the macrochromosome pair 4 of *C. mydas, E. imbricata* and *L. olivacea,* which reinforces the hypothesis of a probable homeology among the pairs and that the morphological differences observed among the chromosomes are the result of a possible centromeric repositioning (Machado *et al*., 2020).

The microsatellite (GA)_n_ showed accumulated signals in the Nucleolus Organizer Region (NOR) locus, except for *E. imbricata*. Studies have shown that the frequent association between microsatellites and NOR regions is explained by large number of microsatellites in rDNA intergenic spacers (Ruiz-Ruano *et al*., 2015; Agrawal and Ganley, 2018). Furthermore, the fact that these microsatellites could be found in blocks, dispersed or absents along the chromosome arms may reflect a possible trend of expansion of these microsatellites in the genomes (Pokorná *et al*., 2011b; Ruiz-Ruano *et al*., 2015). Dispersed distributions of microsatellite on chromosomes are constantly associated with TEs, which can contribute to the dispersion of these sequences in the genome (Pucci *et al*., 2016).

The absence of (CAG)_n_ signals in the chromosomes of *C. caretta* demonstrated that this repetition may have contracted (deletion of repetitive units) in the genome (López-Flores and Garrido-Ramos, 2012) to a reduced number of copies, undetectable by FISH procedure (Kretschmer *et al*., 2018). The number of heterochromatic bands and microsatellites (CA)_n_, (GA)_n_ and (GATA)_n_ sites also were lower in *C. caretta* when compared to *C. mydas*, *E. imbricata* and *L. olivacea*, corroborating that *C. caretta* has a probable divergent karyotype evolution from the other Carettini species.

(GATA)_n_ sequence can be found in the promoter region of the *Doublesex and mab-3-related transcription factor 1* (*Dmrt1*) gene (Raymond *et al*., 2000). *Dmrt1* is a gene that acts during sex determination stages in reptile development (Kettlewell *et al*., 2000; Mizoguchi and Valenzuela, 2020), in which the sex of the embryos is determined by the incubation temperature of the nest (temperature-dependent sex determination: TSD), the most common mechanism of environmental sex determination (ESD) (Ferreira Júnior, 2009). Although, genotypic sex determination was described to have evolved independently in five families of turtles (Chelidae, Emydidae, Geoemydidae, Kinosternidae and Trionychidae) (Valenzuela and Adams, 2011, Valenzuela *et al.*, 2014; Badenhorst *et al*., 2013; Rovatsos *et al*., 2017; Lee *et al*., 2019; Viana *et al*., 2020), sea turtles were described as belonging to a TSD lineage (Bickham *et al*., 1980; Morreale *et al*., 1982; Yntema and Mrosovsky, 1982; Mrosovsky *et al*., 1984, 1992; Valenzuela and Adams, 2011; Montiel *et al*., 2016). Previous *in situ* localization of (GATA)_n_ in three species of turtles with TSD, using both sexes, detected markers preferably in mc and without evidence of sex-specific chromosomal sites (Mazzoleni *et al*., 2019). *In situ* location of (GATA)_n_ in *C. mydas*, *C. caretta*, *E. imbricata* and *L. olivacea* showed accumulations in mc pairs, in addition to a macrochromosome in *E. imbricata*. However, the absence of size heteromorphism between homeologous chromosomes carrying (GATA)_n_ sites reinforces the condition of the absence of heteromorphic sex chromosomes in this group.

In conclusion, this study corroborates the proposal of the centromere repositioning to explain chromosomal morphology alterations in sea turtle diversification, addressing important concerns to absence of pericentromeric heterochromatin in macrochromosomes of Carettini, especially in *C. caretta*. Furthermore, heterochromatic bands and microsatellite sites showed chromosomal differences among *C. mydas*, *C. caretta*, *E. imbricata* and *L. olivacea* karyotypes. These findings encourage further research on the chromosomal evolution in sea turtle species aiming to understand the diversification of Cheloniidae karyotypes.

## Supplementary material

The following online material is available for this article:

Table S1 - Data of the sea turtle species sampled in Brazilian coast or obtained in captive condition.
