# Supplementary material for: Heterochromatin and microsatellites detection in karyotypes of four sea turtle species: Interspecific chromosomal differences
Source: Genet Mol Biol. 2020 Dec 2;43(4):e20200213. doi: 10.1590/1678-4685-GMB-2020-0213 (PMC7734918; doi:10.1590/1678-4685-GMB-2020-0213)
Supplement: Table S1 - [file 1415-4757-GMB-43-4-e20200213-s1.pdf]

Genetics and Molecular Biology
https://doi.org/10.1590/1678-4685-GMB-2020-0213

Supplementary Material to "Heterochromatin and microsatellites detection in karyotypes of four sea turtle species: Interspecific chromosomal differences"

Table S1. Data of the sea turtle species sampled in Brazilian coast or obtained in captive condition. The GPS refers just to place of sample obtention.

Species	Samples	Condition	Localities	GPS
Chelonia mydas	3 juveniles	Captive	Aquário Natal; Extremoz – RN	5º43'53" S; 35º12'16" W
	15 juveniles	Wild	Cobras Island; Pontal do Sul – PR	25º28'55" S; 48º25'56" W
	1 ♀ and 8 juveniles	Captive	Projeto TAMAR; Mata de São João - BA	12º34'38" S; 38º00'07" W
Caretta caretta	1 ♂ and 2 ♀	Captive	Projeto TAMAR; Florianópolis – SC	27º34'19" S; 48º25'40" W
	1 ♂, 3 ♀ and 5 juveniles	Captive	Projeto TAMAR; Mata de São João - BA	12º34'38" S; 38º00'07" W
Eretmochelys imbricata	2 ♀	Wild	Minas Beach; Tibau do Sul – RN	6º14'27" S; 35º02'16" W
	4 juveniles	Captive	Projeto TAMAR; Mata de São João - BA	12º34'38" S; 38º00'07" W
Lepidochelys olivacea	3 juveniles	Captive	Aquário Natal; Extremoz – RN	5º43'53" S; 35º12'16" W
	1 ♂ and 2 juveniles	Captive	Projeto TAMAR; Mata de São João- BA	12º34'38" S; 38º00'07" W

Note: BA = Bahia state; RN = Rio Grande do Norte state; PR = Paraná state; SC = Santa Catarina state.
